# Geostatistical analysis of disease data: estimation of cancer mortality risk from empirical frequencies using Poisson kriging

**DOI:** 10.1186/1476-072X-4-31

**Published:** 2005-12-14

**Authors:** Pierre Goovaerts

**Affiliations:** 1 BioMedware, Inc., Ann Arbor, MI, USA

## Abstract

**Background:**

Cancer mortality maps are used by public health officials to identify areas of excess and to guide surveillance and control activities. Quality of decision-making thus relies on an accurate quantification of risks from observed rates which can be very unreliable when computed from sparsely populated geographical units or recorded for minority populations. This paper presents a geostatistical methodology that accounts for spatially varying population sizes and spatial patterns in the processing of cancer mortality data. Simulation studies are conducted to compare the performances of Poisson kriging to a few simple smoothers (i.e. population-weighted estimators and empirical Bayes smoothers) under different scenarios for the disease frequency, the population size, and the spatial pattern of risk. A public-domain executable with example datasets is provided.

**Results:**

The analysis of age-adjusted mortality rates for breast and cervix cancers illustrated some key features of commonly used smoothing techniques. Because of the small weight assigned to the rate observed over the entity being smoothed (kernel weight), the population-weighted average leads to risk maps that show little variability. Other techniques assign larger and similar kernel weights but they use a different piece of auxiliary information in the prediction: global or local means for global or local empirical Bayes smoothers, and spatial combination of surrounding rates for the geostatistical estimator. Simulation studies indicated that Poisson kriging outperforms other approaches for most scenarios, with a clear benefit when the risk values are spatially correlated. Global empirical Bayes smoothers provide more accurate predictions under the least frequent scenario of spatially random risk.

**Conclusion:**

The approach presented in this paper enables researchers to incorporate the pattern of spatial dependence of mortality rates into the mapping of risk values and the quantification of the associated uncertainty, while being easier to implement than a full Bayesian model. The availability of a public-domain executable makes the geostatistical analysis of health data, and its comparison to traditional smoothers, more accessible to common users. In future papers this methodology will be generalized to the simulation of the spatial distribution of risk values and the propagation of the uncertainty attached to predicted risks in local cluster analysis.

## Background

Because of the need to protect patient privacy publicly available data are often aggregated to a sufficient extent to prevent the disclosure or reconstruction of patient identity. The information available for human health studies is thus often restricted to raw or adjusted rates within areas, such as census units, metropolitan statistical areas, counties, states, and so forth. Associations can then be investigated between these areal data and environmental, socio-economic, behavioral or demographic covariates. Despite the loss of information induced by the aggregation process, the so-called *ecological studies *have some appeal over individual-level observation studies in that they use routinely collected data and typically are carried out over larger geographical areas with greater exposure contrasts [1]. Ideally, the aggregation should not be too coarse to allow a detailed view of geographical patterns in disease incidence. The trade-off cost is however a larger uncertainty or noise in disease data, which is caused by unreliable extreme relative risks estimated over small areas, such as ZIP code areas or census tracts. This effect is known as the "small number problem".

Statistical smoothing algorithms have been developed to filter local small-scale variations from mortality maps, enhancing larger-scale regional trends [2,3]. These methods greatly differ in their computer requirements, as well as underlying assumptions regarding the spatial patterns and distribution of risk values. The most straightforward smoothers are deterministic and involve a simple weighted average of neighboring rates. The kernel weights can be based, for example, on the inversed squared distance to the area being smoothed and/or the population size of those areas [4]. The median-based head banging smoother takes into account both the spatial geometry and the values of the surrounding observations; weighted versions incorporate the variance of the rates to account for the lack of reliability of rates computed from small populations [5]. A limitation of such simple smoothers is that they are not easily tailored to the pattern of variability displayed by the data. For example, important features such as anisotropy (i.e. direction-dependent variability) and the range of spatial correlation are not accounted for by the inverse squared distance method. Another important weakness is that, in absence of any probabilistic modeling, the uncertainty attached to the smoothed rates cannot be quantified.

Model-based approaches treat the observed response, i.e. number of deaths or cases, as the realization of a random variable with a specific probability distribution (i.e. Poisson or binomial random variable). Over the years, statisticians have developed models of increasing complexity, combining fixed effects with both uncorrelated and spatially structured random effects, leading to mixed effects or hierarchical models [6-10]. Most of these methods have been developed within a Bayesian framework whereby the terms in the model are assigned prior distributions that, in turn, have "hyperprior" parameters. Full Bayesian modeling assigns prior distributions to these hyperparameters, which allows all sources of uncertainty in the model to be taken into account. The trade-off cost for the flexibility of a full Bayesian approach is the complexity of the estimation of model parameters. This step is performed using iterative procedures, such as Markov Chain Monte Carlo (MCMC) methods, that are computer intensive and require fine-tuning, which makes their application and interpretation challenging for non-statisticians [11,12]. Empirical Bayes methods simplify greatly the estimation procedure by assigning point estimates (i.e. obtained by maximum likelihood [13] or method of moment [14] from the data) to the hyperparameters. Although the empirical methods neglect the variability associated with the parameter estimation and allow only computation of approximate standard errors for the risk, they are easier to implement and are favored by practitioners.

Probabilistic modeling of aggregated health data has also been conducted in the geostatistical literature, outside the mainstream of health statistics. Geostatistics provides a set of statistical tools for the analysis of data distributed in space and time. It allows the description of spatial patterns in the data, the incorporation of multiple sources of information in the mapping of attributes, the modeling of the spatial uncertainty and its propagation through decision-making [15,16]. Since its development in the mining industry, geostatistics has emerged as the primary tool for spatial data analysis in various fields, ranging from earth and atmospheric sciences, to agriculture, soil science, environmental studies, and more recently exposure assessment and environmental epidemiology [17]. The traditional implementation of geostatistical methods however does not accommodate the heteroscedasticity of disease rates and counts, i.e. the fact that their variance in each place varies as a function of the population size [18]. Alternatives to the Matheron's semivariogram estimator and kriging algorithms thus need to be developed to account for the specific nature of health data.

In the geostatistical literature one finds three main approaches to account for the problem of non-stationarity of the variance caused by spatially varying populations. The first solution, which is the most straightforward to implement, is to transform the rates before conducting a classical geostatistical analysis. In his book (p.385–402), Cressie [19] proposed a two-step transform of the data to remove the mean-variance dependence of the data and the heteroscedasticity. Traditional variography was then applied to the transformed residuals. Berke [20] described an empirical approach whereby rates are smoothed using global empirical Bayes estimation before being interpolated using kriging. Despite its simplicity, Berke's approach suffers from several drawbacks, such as the inability to account for the uncertainty attached with the transformed rates, the aspatial nature of the transform, and the oversmoothing caused by the combination of Bayes smoothing and kriging.

Another approach is to incorporate the impact of population size directly into the semivariogram inference and spatial interpolation. To attenuate the influence of unreliable rates in the modeling of spatial variability, Goovaerts [21,22] proposed a population-weighted semivariogram estimator. The noise caused by small population sizes is then filtered from the raw rates by a variant of factorial kriging where the kriging weights are rescaled *a posteriori *using the population size of each observation. Although this approach is relatively straightforward and improves over simple population-weighted estimators as illustrated by extensive simulation studies, there are a few limitations. First, the algorithm filters together the variability arising from data reliability (spatially varying population size) and the potential noise of the underlying risk. Second, the *a posteriori *rescaling is empirical and might affect the optimum properties of the kriging estimator.

The most rigorous, yet mathematically challenging, solution is to derive new semivariogram estimator and kriging algorithms, taking into account the binomial or Poisson nature of the count data. The first initiative must be credited to Oliver *et al. *[23] who studied the risk of childhood cancer in the West Midlands of England. They developed an approach that accounts for spatial heterogeneity in the population of children to estimate the semivariogram of the "risk of developing cancer" from the semivariogram of observed mortality rates. Binomial cokriging was then used to produce a map of cancer risk. Goovaerts [24] proposed a variant of binomial cokriging that is more flexible and robust with respect to misspecification of the underlying hypothesis. Simulation studies conducted under different spatial patterns of risk and population size scenarios demonstrated that the combined use of population-weighted semivariogram and rescaled cokriging system leads to more accurate estimates of the underlying risk than the traditional implementation of binomial cokriging or a simple population-weighted local mean. The approach also outperformed empirical Bayes smoothers in the majority of cases. In another study on female breast cancer in Long Island, New York, the rates smoothed by the binomial cokriging variant were used in a local cluster analysis, leading to the detection of larger and more compact clusters of low or high rates as well as the disappearance of some unreliable spatial outliers recorded over sparsely populated ZIP codes [25].

More recently, Monestiez *et al. *[26,27] developed Poisson kriging for mapping the relative abundance of species in the presence of spatially heterogeneous observation efforts and sparse animal sightings. In this case the denominator was the observation time or effort. This filtering approach is similar to the one developed by Oliver *et al*., except that a Poisson distribution replaces the Binomial distribution for counts and therefore is consistent with the assumption underlying the development of empirical Bayes smoothers. The estimation of the experimental semivariogram of the risk is also more robust since there is no need to adopt an iterative procedure to estimate the variance of the risk. In their paper, Monestiez *et al. *compared Poisson kriging with Diggle *et al*.'s "model-based kriging" which is in fact a Generalized Linear Mixed Model (GLMM) where the random effect is a spatial Gaussian process [28]. Poisson kriging was 500 times faster than GLMM since it does not require lengthy iterative procedure for parameter estimation. It is also more flexible since it avoids the subjective modeling of the risk values as a lognormal random field. Both methods yielded equivalent results for 90% of the predictions and differed only for higher values with a smoothing for the kriging. They also found that the lognormal hypothesis induced similarities between the maps of the GLMM prediction variance and estimate (i.e. proportionality), while the Poisson kriging variance mainly reflects the observation effort (i.e. lower variance for longer observation times).

The Poisson kriging approach was generalized by Goovaerts to the analysis of disease data; in particular the detection of disparities in prostate cancer mortality between black and white males over the continental US and five 5-yr periods [29]. Prediction performance of Poisson kriging was however not quantified and a comparison to other common smoothers was also lacking. The objective of this paper is to introduce Poisson kriging for the analysis of disease data and describe a public-domain executable that allows automatic computation and modelling of risk semivariograms, followed by the estimation of risk values at sampled locations. Simulation studies are conducted to compare the performances of the new methodology to other readily accessible smoothers (i.e. population-weighted estimators and empirical Bayes smoothers) under different scenarios for the disease frequency, the population size, and the spatial pattern of risk. Performance criteria include the accuracy of the prediction of the underlying risk and the quantification of the attached uncertainty.

## Methods

### Data

**Figure 1:**
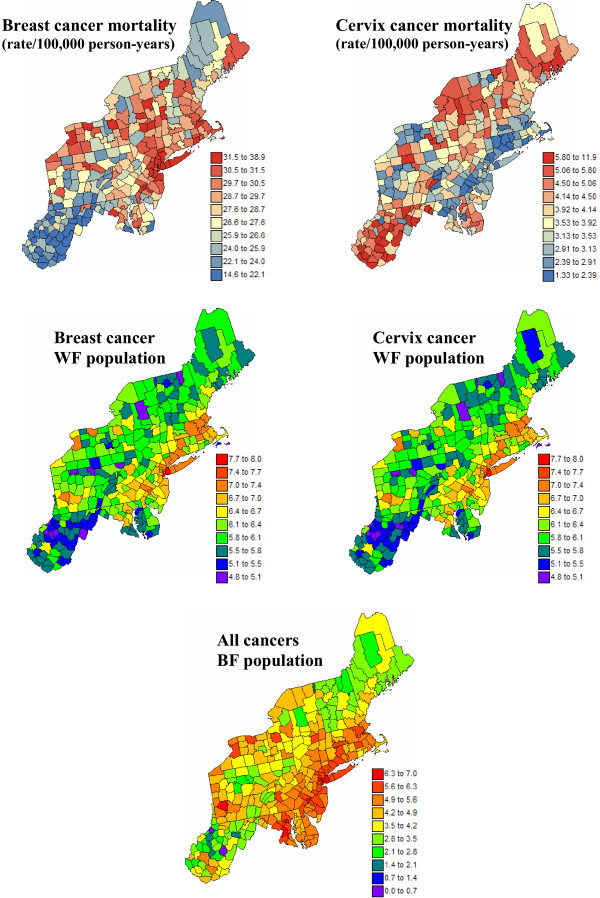
**Geographic distribution of age-adjusted breast and cervix cancer mortality rates and the populations at risk**. For the two top maps, the fill color in each county represents the age-adjusted mortality rates per 100,000 person-years recorded over the period 1970–1994 for white females (class boundaries correspond to deciles of the histogram of rates). The middle maps represent the population at risk which was back-calculated from the rate and count data (lognormal scale). The bottom map represents the population at risk for black females (BF) which was back-calculated from the "all cancers" age-adjusted rate and the count data.

The use of Poisson kriging for the analysis of health data will be illustrated using directly age-adjusted mortality rates for a frequent (i.e. breast) and less frequent (i.e. cervix) cancer. These data are part of the Atlas of Cancer Mortality in the United States [30] and were downloaded from . The rates were adjusted using the 1970 population pyramid. The analysis focuses on white female rates recorded over the 1970–1994 period for 295 counties of 12 New England States. Figure 1 (top graphs) shows, for both cancers, the spatial distribution of age-adjusted mortality rates per 100,000 person-years. Following the recommendations of several studies on map color schemes [31,32], a double-ended color scheme with 10 equally-weighted classes (i.e. boundaries correspond to deciles of the histogram) was used: a gradient of red is used for rates higher than the median, while a gradient of blue is used for lower rates. The two cancers display opposite spatial patterns: higher breast cancer mortality rates along the East Coast (Baltimore to Boston) and lower rates in the Southern part of the study area, and the reverse for cervix cancer. For each cancer, the population at risk was computed as: 100,000 × the total number of deaths over the 1970–1994 period divided by the corresponding age-adjusted mortality rate; both datasets are available on NCI website. The population sizes are mapped in Figure 1 (middle row) using a rainbow color scheme to avoid any confusion with the rate maps. The population-weighted average of the age-adjusted cancer mortality rates is 30.14 per 100,000 person-years for breast and 3.23 per 100,000 person-years for cervix.

Multiple realizations of the spatial distribution of cancer rates will be simulated from the maps of mortality rates and population sizes using the procedure described in the results and discussion section. To investigate how the various algorithms perform under highly unstable rates, simulations will also be conducted using the population at risk for black females. These populations were computed as: 100,000 × the total number of "all cancers" deaths over the 1970–1994 period divided by the age-adjusted mortality rate. The "all cancers" category was used to avoid zero population estimates caused by the small minority population, and corresponding missing rate data, in many of these counties. The minority population map is displayed at the bottom of Figure 1.

### Poisson model for rare diseases

For a given number *N *of entities (e.g. counties, states, electoral ward), denote the number of recorded mortality cases by d(**u**_α_) and the size of the population at risk is n(**u**_α_). Following most authors entities are referenced geographically by their centroids (or seats) with the vector of spatial coordinates **u**_α _= (x_α_, y_α_), which means that the actual spatial support (i.e. size and shape of the county or ward) is ignored in the analysis. Future research outlined in the conclusions seeks to relax this assumption which is unsatisfactory when working with vastly different entities, such as SEA units over the US. The empirical or observed mortality rates are then denoted as z(**u**_α_) = d(**u**_α_)/n(**u**_α_).

At each location **u**_α_, the disease count d(**u**_α_) can be interpreted as a realization of a random variable D(**u**_α_) that follows a Poisson distribution with one parameter (expected number of counts) that is the product of the population size n(**u**_α_) by the local risk R(**u**_α_):

D(**u**_α_) | R(**u**_α_) = Poisson(n(**u**_α_)R(**u**_α_)) α = 1,...,N     (Equation 1)

Given the risk value R(**u**_α_), the count variables D(**u**_α_) are assumed to be conditionally independent. In other words, any spatial correlation among the counts is caused by spatial trends in either the population sizes or in the local individual risks. The risk variable R(**u**) itself can be modeled as a stationary random field with mean m, variance  and covariance function C_R_(**h**).

The conditional mean and variance of the rate variable Z(**u**_α_) are defined as:

E[Z(**u**_α_) | R(**u**_α_)] = R(**u**_α_)     (Equation 2)

Var[Z(**u**_α_) | R(**u**_α_)] = R(**u**_α_)/n(**u**_α_)     (Equation 3)

Following Waller and Gotway [17], the unconditional mean and variance are as follows:

E[Z(**u**_α_)] = E[R(**u**_α_)] = m     (Equation 4)

Var[Z(**u**_α_)] = Var[R(**u**_α_)] + E[R(**u**_α_)/n(**u**_α_)] =  + m/n(**u**_α_)     (Equation 5)

Different methods are available to estimate the risk over a given entity with centroid **u**_α _from the set of observed rates, {z(**u**_α_), α = 1,...N}. The estimators introduced in this paper can all be formulated as a linear combination of neighboring rates or functions of those rates. Neighbors can be selected in a number of ways, such as those areas having centroids within a specified distance of the target entity's centroid **u**_α _[3] or those that share a border with the area to be smoothed [14]. These subjective neighborhood definitions can impact the analysis, particularly when areas vary greatly in size and shape [18]. In this paper and the attached program, the search strategy allows the user to select a maximum number of neighbors that fall within a fixed distance from the centroid of the area to be smoothed. This approach is flexible enough to handle change in the size of geographical units, hence change in the spatial density of centroids, across the study area. The search strategy is further discussed in the results and discussion section

### Traditional smoothers

#### Population-weighted average

A straightforward estimate of the risk at location **u**_α _is a population-weighted average (PWA, see [17] p. 87) of K neighboring observed rates:



where λ_*i*_(**u**_α_) is the weight assigned to the rate observed at **u**_i _when predicting the risk at location **u**_α_. Although the PWA estimator is not derived under a stochastic model, an approximate mean square error of prediction can be computed using an approach similar to the computation of the error variance in geostatistics [[15], p. 128]. Assuming that the estimator is unbiased, the mean square error of prediction is computed as:



where C(**u**_i _- **u**_j_) is the covariance between the rates measured at locations **u**_i _and **u**_j_, and C(**u**_i _- **u**_α_) is the covariance between the rate measured at location **u**_i _and the unknown risk at **u**_α_. A simplifying assumption is that these two spatial covariance functions are the same. In this paper, that function is derived as C(**h**) = C(0)-γ(**h**), where C(0) is the sill of the semivariogram model γ(**h**) fitted to the following population-weighted semivariogram [24]:



where N(**h**) is the number of data pairs separated by the vector **h**. The fitting of the model to the experimental semivariogram values is performed using the weighted least-square algorithm described in later section on the public-domain executable.

#### Empirical Bayes smoothers

In Empirical Bayes estimation, the risk r(**u**_α_) is computed as a weighted sum of the rate observed at that location (kernel rate) and a prior mean that can be either global or local. Following the method of moments proposed by Marshall [14], the global Bayes smoother of the rate at **u**_α _is as follows:



The Bayes shrinkage factor λ(**u**_α_) is computed as:



where m* and s^2 ^are the population-weighted sample mean and variance of rates, and  is the average population size across the study area. Whenever the rate z(**u**_α_) is based on small population sizes n(**u**_α_) relative to the average size , the factor λ(**u**_α_) is small and the Bayesian estimate (Equation 9) is close to the global mean m*. In other words, the relative weight assigned to the observed rate is small since it is deemed less reliable. This weight is further reduced if the variance of the rates, s^2^, decreases; that is if the spatial homogeneity of the observed rates increases. In the extreme situation where the variance s^2 ^is lower than the ratio m*/, the smoothed risk map is uniform with  (**u**_α_) = m* ∀ **u**_α_.

Assuming that the shrinkage factor is known, an approximate mean square error of prediction can be computed as:



The mean square error of prediction by the global mean rate at **u**_α_,  (**u**_α_), is computed by applying an expression similar to Equation 7 to the entire set of N rates:



where n_tot _is the sum of the at-risk population over all N entities.

**Figure 2:**
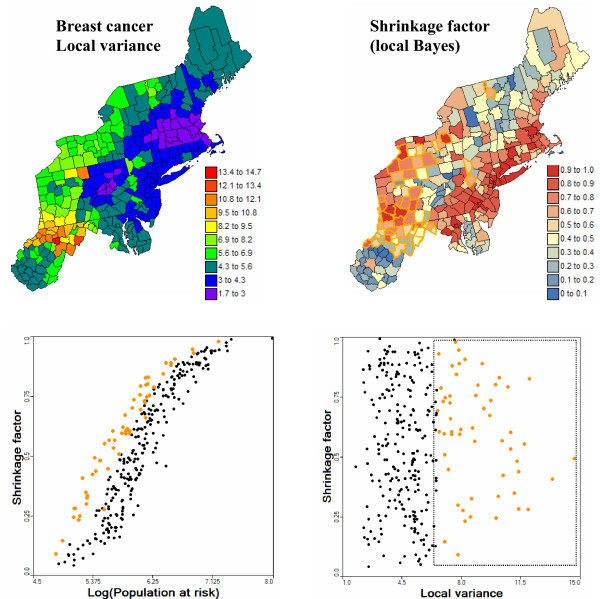
**Impact of population size and local variance on the shrinkage factor in empirical Bayes smoothing**. The local variance is the population-weighted variance among the 32 closest breast cancer mortality rates for each county. The shrinkage factor represents the relative weight assigned to the local rate versus the local mean in the local empirical Bayes smoother. This factor increases with the population size of the county (i.e. more reliable rate) and in regions where the local variance is high. Selected counties are depicted by orange dots in the scattergram and orange boundaries on the map of shrinkage factor.

The global empirical estimator (Equation 9) is spatially invariant: any rearrangement of the geographical entities leaves the estimates unchanged. An alternative, which accounts for the fact that areas that are close to each other tend to have similar rates, is to consider a prior mean for each area such that the estimated risks are shrunk towards local means instead of a general mean. Local Bayes smoothers are computed similarly except that all the statistics (i.e. the population-weighted sample mean and variance, population size) are computed within local search windows [14]; for example using the K neighboring observed rates. The estimator thus becomes:



where m* (**u**_α_) =  (**u**_α_). The Bayes shrinkage factor λ(**u**_α_) is now computed as:



As for global Bayes smoothers, the relative weight λ(**u**_α_) assigned to the observed rate z(**u**_α_) is smaller for less densely populated counties. For counties with similar population sizes, the factor λ(**u**_α_) is also smaller in areas of greater homogeneity, as measured by a lower local variance s^2^(**u**_α_). In other words, where there is less local variability, the estimate tends to be closer to the local mean. For example, Figure 2 shows the maps of the local variance s^2^(**u**_α_) and the shrinkage factor λ(**u**_α_). The left bottom scattergram illustrates the positive relationship between the shrinkage factor and the population size. The counties highlighted in orange correspond to higher local variance (see right scattergram), and these counties are associated with higher λ(**u**_α_) values for similar population sizes.

By analogy with the global Bayes smoother, the mean square error of prediction can be computed as:



### Poisson kriging

In Poisson kriging, the risk over a given entity with centroid **u**_α _is estimated as the following linear combination of *K *neighboring observed rates:



Unlike the population-weighted average (Equation 6), the weights λ_*i*_(**u**_α_) are here computed so as to minimize the mean square error of prediction under the constraint that the estimator is unbiased. These weights are the solution of the following system of linear equations, known as the "Poisson Kriging" (PK) system [26,27]:



where δ_ij _= 1 if **u**_i _= **u**_j _and 0 otherwise, and m* is the population-weighted mean of the rates. The term μ(**u**_α_) is a Lagrange parameter that results from the minimization of the estimation variance subject to the unbiasedness constraint on the estimator. The addition of an "error variance" term, m*/*n*(**u**_i_), for a zero distance accounts for variability arising from population size, leading to smaller weights for less reliable data (i.e. measured over smaller populations). This term actually corresponds to the difference between the variances of the risk and rate variables, recall the expression for the unconditional variance (Equation 5). Note that kriging is used here to filter the noise from the observed rates aggregated to the county level, not to estimate the risk within the unit itself (disaggregation procedure). There is no change of support and the underlying hypothesis is that all counties have the same spatial support.

The prediction variance associated with the estimate (15) is computed using the traditional formula for the ordinary kriging variance:



This statistic differs, however, from the traditional kriging variance in that: 1) it depends not only on the data configuration but also on the reliability of each of these data which is a function of the population size, and 2) the kriging variance is non-zero even when estimating at a sampled location since the quantity to be estimated (risk) is different from the one measured (empirical rate). The computation of kriging weights and kriging variance (Equations (16) and (17)) requires knowledge of the covariance of the unknown risk, *C*_*R*_(**h**), or equivalently its semivariogram γ_*R*_(**h**) = *C*_*R*_(0)-*C*_*R*_(**h**). Following Monestiez *et al. *[26,27] the semivariogram of the risk is estimated as:



where the different pairs [*z*(**u**_α_)-*z*(**u**_α _+ **h**)] are weighted by the corresponding population sizes to homogenize their variance.

### Public-domain executable

**Figure 3:**
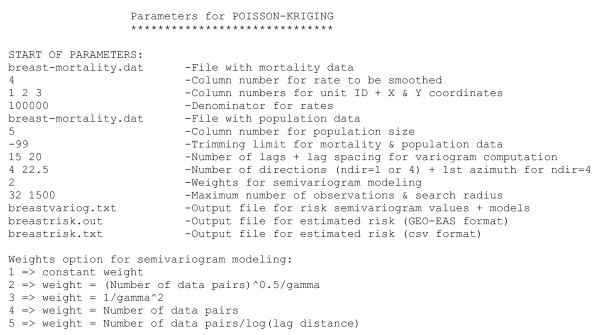
**Example of parameter file required by poisson_kriging.exe**. This parameter file is used to conduct a geostatistical analysis of breast cancer mortality rates displayed in Figure 1. Semivariograms are computed using 15 classes of 20 km, in four directions with the first direction azimuth starting at 22.5° measured clockwise from the NS axis.

**Figure 4:**
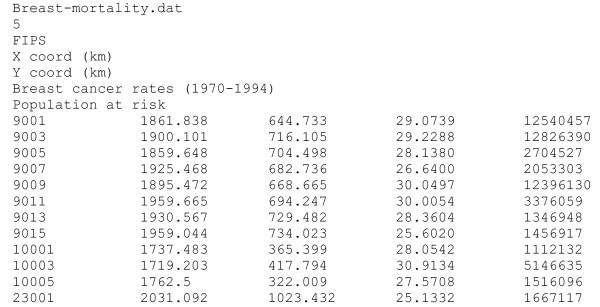
**Example of dataset for poisson_kriging.exe**. Data for the Poisson kriging program must be in Geo-EAS format. The first line is the name of the data file. The second line should be a numerical value specifying the number of variables (i.e. nvar columns) in the data file. The next *nvar *lines contain the name of each variable. The following lines, until the end of the file, are considered as observations and must have *nvar *numerical values per line.

To disseminate the use of this new methodology an executable was developed and is provided with the paper (additional file 1: poisson-kriging.exe), along with a sample dataset (additional file 2: breast-mortality.dat) and parameter file (additional file 3: poisson-kriging.par). The source code was built around the Gslib kriging program KT3D [33] and the semivariogram modelling program VARFIT [34]. Like these two public-domain programs, the Poisson kriging source code is written in ANSI standard Fortran 77 and was compiled on a PC. The reader interested in having a version compiled on another operating system should contact the author. When running the executable, called **poisson_kriging.exe**, the user needs to specify the name of a parameter file that includes all the variables and names of input/output files required by the program. A typical parameter file, which was used to analyze breast cancer data, is illustrated in Figure 3. The text file, called **poisson-kriging.par**, includes the following information:

• Name of the text file including the mortality dataset. This dataset must be in Geo-EAS format [35]. An example for the file **breast-mortality.dat **is given in Figure 4. The first line is the name of the data file. The second line should be a numerical value specifying the number of variables (i.e. *nvar *columns) in the data file. The next *nvar *lines contain the name of each variable. The following lines, until the end of the file, are considered as observations and must have *nvar *numerical values per line (the program has been compiled to read a maximum of 50,000 observations). For example, the 1^st ^observation corresponds to Fairfield county (CT, FIPS code 9001). The spatial coordinates of its geographic centroid are X = 1861.838 km and Y = 644.733 km. The US Albers Equal Area projection was used in this study. The age-adjusted breast cancer mortality rate is 29.0739 per 100,000 person-years and the population at risk for the 25-yr period is 12540,457.

• The column number for the mortality rate.

• The column numbers for the observation identification code (i.e. FIP county), and the variables with the spatial coordinates.

• The denominator for the rate (i.e. 100,000 persons for the data from the cancer mortality Atlas).

• Name of the text file including the population dataset.

• The column number for the population at risk.

• Trimming limit. All mortality rates and population sizes lesser or equal to that value are ignored in the analysis. A risk value is however estimated at each location (i.e. local and global means for the local and global empirical Bayes smoothers).

• Number and width of classes of distances used for the computation of the semivariogram; 15 classes of 20 km are used in this study.

• Number of directions for the computation of the semivariogram. Options are *ndir *= 1 (omnidirectional) and *ndir *= 4. In the later case, the semivariogram is computed in four directions (angular tolerance = ± 22.5°), starting with the azimuth direction specified by the user. Using the Gslib convention [33], angles are measured in degrees clockwise from the NS direction.

• Weighting scheme used in the least-square fitting of a semivariogram model to experimental values. The program will try all possible combinations of 1 or 2 basic models from the following three permissible semivariogram models: spherical, exponential and cubic. The selected model is the one that minimizes the weighted sum of squares of differences between the experimental and model curves:



where *L *is the number of classes of distance. The user can choose among the five following types of weighting schemes: w(**h**_l_) = 1, w(**h**_l_) = /γ(**h**_l_), w(**h**_l_) = 1/γ(**h**_l_)^2^, w(**h**_l_) = N(**h**_l_), w(**h**_l_) = N(**h**_l_)/log|**h**_l_|. Except for the first option, each alternative set of weights aims to assign more importance to: semivariogram values computed from many data pairs (hence more reliable), and/or smaller semivariogram values that are typically observed for short distances, since the behavior of the semivariogram at the origin has the largest impact on kriging results.

**Figure 5:**
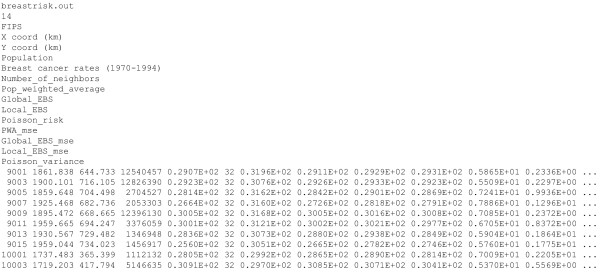
**Output file created by poisson_kriging.exe following the analysis of breast cancer mortality rates**. The output file (Geo-EAS format) includes the estimated risk values and associated mean square errors of prediction for the following predictors: population-weighted average, global and local Bayes smoothers, Poisson kriging. This output file was obtained when running the code **poisson_kriging.exe **with the parameter file of Figure 3.

• Maximum number of neighboring observed rates, K, to be used in the estimation, and maximum size of the search window. In this example, a large radius of 1,500 km is chosen so as to guarantee that across the study area 32 observations are always found within the search window.

• Name of output text file reporting the experimental semivariogram values and the parameters (i.e. type of basic model, nugget effect, sill, range, anisotropy angle) of the model fitted. Three estimators are used: traditional semivariogram (Equation 8 with n(**u**_α_) = 1 ∀ α), population-weighted semivariogram (Equation 8), and risk semivariogram (Equation 18).

• Name of output text file (Geo-EAS format) that includes the estimated risk values and associated mean square errors of prediction for the following predictors: population-weighted average, global and local Bayes smoothers, Poisson kriging. The number *K *of neighbors used in the estimation is also reported. An example for the breast cancer data, file **breastrisk.out**, is given in Figure 5.

• Name of output text file (csv format) that includes the same information as the file **breastrisk.out **but in a format (comma delimited) that can be easily imported into Excel.

**Figure 6:**
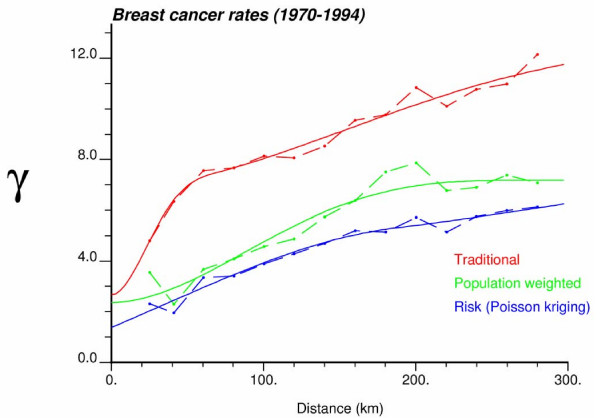
**Three omnidirectional semivariograms of breast cancer mortality rates with the model fitted**. This graph was created by **poisson_kriging.exe **using the parameter file of Figure 3 with the option *ndir *= 1. The estimators are the followings: traditional semivariogram (Equation 8 with n(**u**_α_) = 1 ∀ α), population-weighted semivariogram (Equation 8), and risk semivariogram (Equation 18). The solid curve denotes the model fitted using weighted least-square regression.

**Figure 7:**
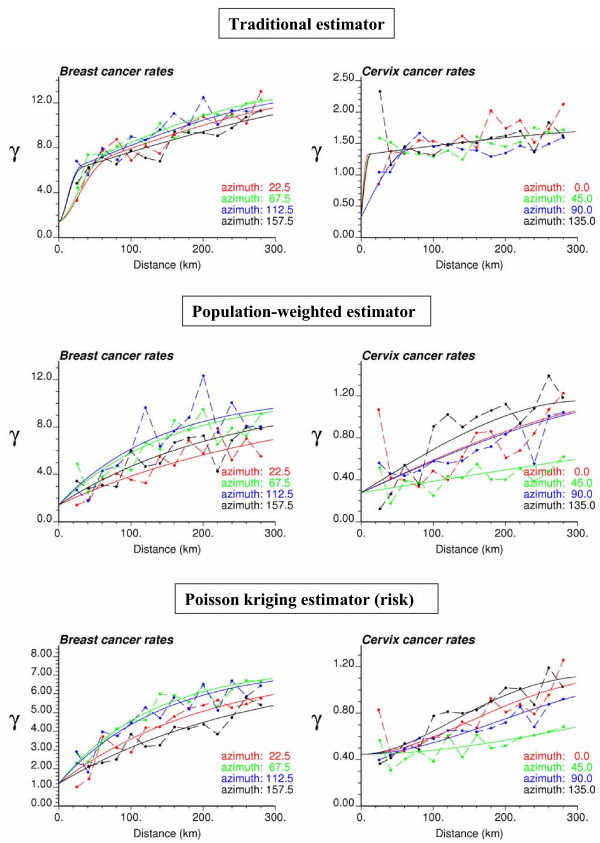
**Directional semivariograms for breast and cervix cancer mortality rates with the anisotropic model fitted**. The graphs for breast cancer (left column) were created by **poisson_kriging.exe **using the parameter file of Figure 3 with the option *ndir *= 4. The estimators are the followings: traditional semivariogram (Equation 8 with n(**u**_α_) = 1 ∀ α), population-weighted semivariogram (Equation 8), and risk semivariogram (Equation 18). The semivariograms are computed in four directions; azimuth angles are measured in degrees clockwise from the NS axis The solid curve denotes the anisotropic (i.e. direction-dependent) model fitted using weighted least-square regression.

In addition to text files with the estimated risk values, **poisson_kriging.exe **generates graphs that display the experimental semivariogram values and the model fitted. These figures are in PostScript format and can be viewed using the public-domain program GSview . These graphs should help detecting any poor choice of the number and width of classes of distances, as well as poor fits of semivariogram models. In the later case, the user should select other options for the weighting scheme. For the option *ndir *= 1, all omnidirectional semivariograms are plotted on the same graph, called **all-variog.ps**; see example for breast cancer in Figure 6. When directional semivariograms are computed (*ndir *= 4), three postscript files will be created for the traditional (**trad-variog.ps**), population-weighted (**weighted-variog.ps**), and risk semivariogram estimators (**risk-variog.ps**). Figure 7 (left column) shows an example for breast cancer rates.

## Results and discussion

### Analysis of breast and cervix cancer data

Mortality risks for breast and cervix cancers were estimated from the age-adjusted mortality rates displayed at the top of Figure 1 using the four alternative methods: population-weighted average (PWA), local (LBS) and global (GBS) empirical Bayes smoothers, and Poisson kriging (PK). All local statistics were computed using K = 32 closest observations which were selected according to the Euclidian distance between the county geographical centroids. The impact of the parameter *K *on the results was investigated by repeating the analysis with 16 and 64 observations. Fewer observations led to larger prediction errors while using more observations increased the smoothing of the original rates. The following discussion is thus limited to the choice K = 32.

Figure 7 shows the semivariograms computed for both types of cancer in the four main directions (azimuth are measured in degrees clockwise from the NS axis). The three estimators (traditional, population-weighted, and risk) implemented in **poisson_kriging.exe **are displayed. On each graph, the solid curve denotes the model fitted using weighted least-square regression. For example, the traditional estimator for breast cancer was modeled using a combination of a cubic model (min. range = 36 km, max. range = 215 km) and spherical model (min. range = 359 km, max. range = 530 km). The two other semivariograms for breast cancer were modeled using an exponential model: population-weighted estimator (min. range = 411 km, max. range = 1198 km), and risk semivariogram (min. range = 381 km, max. range = 947 km).

Accounting for population sizes (estimators of Equations 8 and 18) attenuates the impact of data pairs that involve at least one rate computed from small populations, revealing structures that might be blurred by the random variability of extreme values. This effect is more pronounced for cervix cancer: the population-weighted and risk semivariograms are better structured and display much longer ranges of autocorrelation than the traditional estimator that indicates a very weak spatial correlation (i.e. almost pure nugget effect). Since cervix cancer is less frequent than breast cancer, its mortality rates are more likely to be impacted by the small number problem and display higher levels of noise. The weighting also tends to lower the sill of the semivariogram which represents the spatial variance of mortality rates. This decrease is particularly clear on the plot of all omnidirectional semivariograms in Figure 6. The anisotropy is more pronounced for cervix cancer, with a better spatial continuity (i.e. lower variance) along the NE-SW direction (green semivariogram curve). Slightly better continuity for breast cancer is observed along the NS direction.

**Figure 8:**
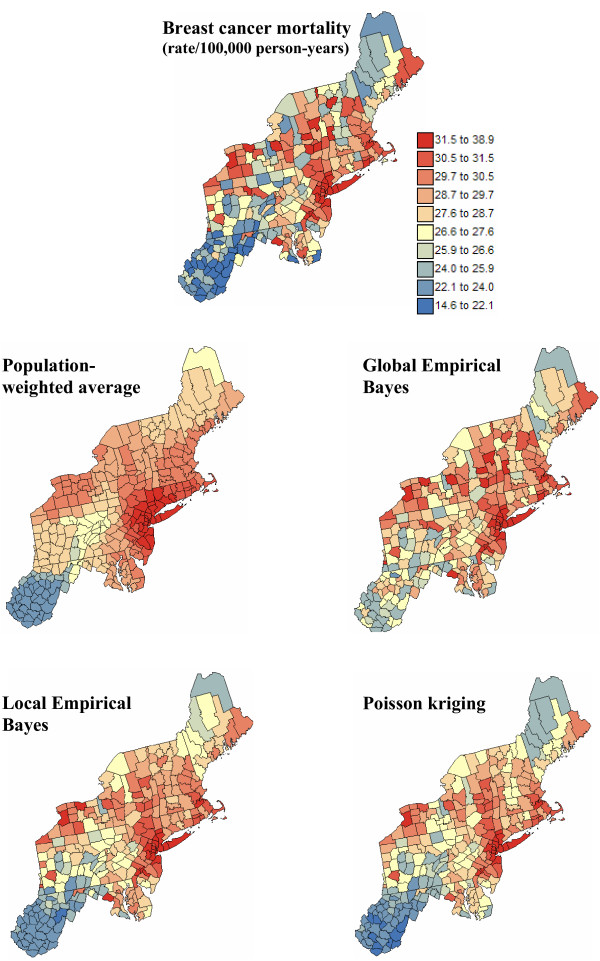
**Maps of age-adjusted breast cancer mortality rates and the risk computed using alternative estimators**. The fill color in each county represents the age-adjusted mortality rate per 100,000 person-years recorded over the period 1970–1994 (top graph) or the risk estimated using the following approaches: population-weighted average, global and local empirical Bayes smoothers, and Poisson kriging. The color legend applies to all the maps; the class boundaries correspond to the deciles of the histogram of original rates.

**Figure 9:**
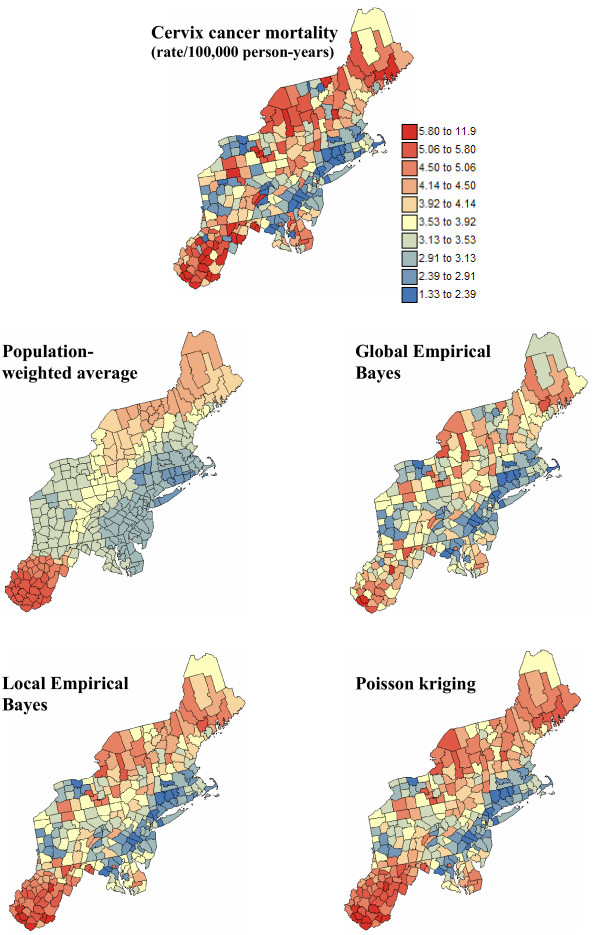
**Maps of age-adjusted cervix cancer mortality rates and the risk computed using alternative estimators**. The fill color in each county represents the age-adjusted mortality rate per 100,000 person-years recorded over the period 1970–1994 (top graph) or the risk estimated using the following approaches: population-weighted average, global and local empirical Bayes smoothers, and Poisson kriging. The color legend applies to all the maps; the class boundaries correspond to the deciles of the histogram of original rates.

Figures 8 and 9 show the maps of risk estimates for breast and cervix cancers. Table 1 indicates that, on average over the 295 counties, the mortality risks estimated by the four methods are very close to the observed rates. Poisson kriging has the closest agreement between average risk and rate. The variance of the estimated risk values is, however, at least half the variance of observed rates. Population-weighted average and the global Bayes smoother generate the largest smoothing effect. Although these two sets of risk estimates have similar variances, their distribution in space is very different; see middle row in Figures 8 and 9. The map of PWA risk values looks much more continuous in space, which is caused by the smaller weight assigned to the kernel rate in the estimation window. In other words, among the 32 mortality rates used to compute the risk value for any given county, the average weight allocated to the rate from that county (kernel weight) is one order of magnitude smaller for PWA (0.03 ≈ 1/32) than for other estimators; see Table 1.

**Table 1:** Summary statistics for breast and cervix cancer risk estimates. Mean and variance of observed age-adjusted mortality rates per 100,000 person-years and risk values estimated using four alternative methods. The average weight assigned to the original rate in the estimation of the risk at the same location is reported as "kernel weight".

Estimator	Breast cancer			Cervix cancer		
	Mean	Variance	Kernel weight	Mean	Variance	Kernel weight

Observed rates	27.31	14.99	1.00	4.02	2.02	1.00
Population-weighted average	28.61	5.89	0.03	3.61	0.50	0.03
Global Empirical Bayes	28.65	4.44	0.68	3.63	0.61	0.66
Local Empirical Bayes	28.02	7.54	0.55	3.83	0.91	0.61
Poisson kriging	27.62	8.00	0.51	3.96	0.95	0.60

**Figure 10:**
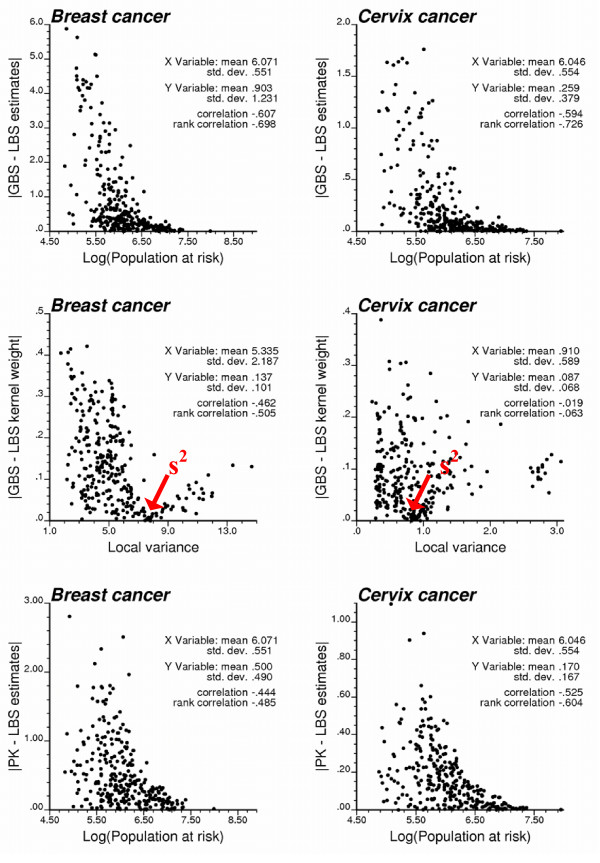
**Impact of population size and local variance on differences between estimates of cancer mortality risk**. Top and bottom scattergrams illustrate the largest differences among mortality risks estimated for less densely populated counties. Middle row shows that the local and global empirical Bayes smothers yield similar shrinkage factor (kernel weight) for areas where the local variance s^2^(**u**_α_) is close to the global variance s^2^.

Because the global empirical Bayes smoother "shrinks" values towards the global mean, the corresponding risk maps are patchier than the ones produced by other estimators that account for the locally varying mean of mortality rates. The scattergrams at the top of Figure 10 show that the discrepancy between LBS and GBS estimates increases as the population size decreases. In less densely populated counties, rates are strongly shrunk towards either the local or the global mean of the rates. Discrepancies also increase as the local mean diverges from the global mean; for the example of breast cancer, the LBS estimates are much smaller than GBS values in the Southern part of the study area characterized by lower age-adjusted mortality rates. In addition to the population size, increasing differences between the local and global variances, s^2^(**u**_α_) and s^2^, tend to inflate differences between the kernel weights (i.e. shrinkage factor) computed by the two types of empirical Bayes smothers. This effect is particularly clear for breast cancer; see Figure 10 (middle row).

**Figure 11:**
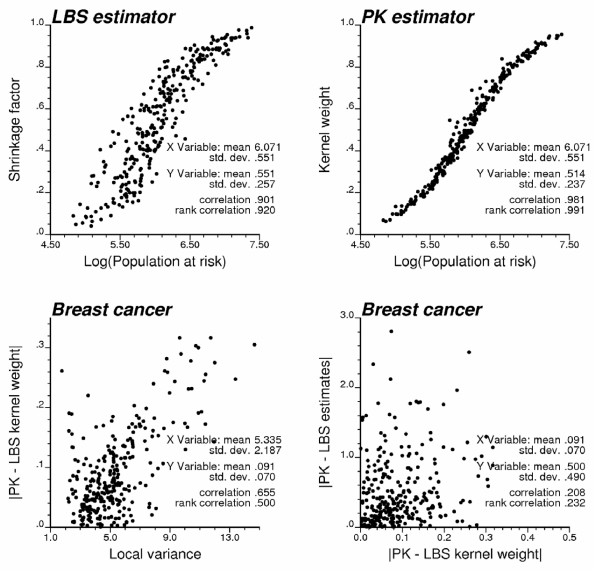
**Impact of population size and local variance on LBS and PK results**. The top scattergrams illustrate the increasing weight assigned to the original mortality rate (i.e. shrinkage factor or kernel weight) as the population in that county increases. The local empirical Bayes (LBS) and Poisson kriging (PK) weights are more distinct in zones of greater variability in mortality rates, as measured by the local variance. Differences between kernel weights are not correlated with the differences between the corresponding risk estimates.

The risk maps generated by the local empirical Bayes smoother and Poisson kriging share the most similarities for cervix cancer, and in the Southern part of the region for breast cancer. Like for the two types of empirical Bayes smoothers, the difference between Poisson kriging and LBS estimates increases as the population size of the county decreases, see Figure 10 (bottom graphs). As the mortality rate becomes less reliable, more weight is assigned to other pieces of information (i.e. global mean for GBS, local means for LBS, or surrounding observations for PK), leading to larger deviations between estimators. Another difference lies in the weight assigned to the mortality rates (i.e. kernel weight) in the computation of the PK and LBS estimates. Figure 11 (top graphs) shows that the relationship between population size and kernel weight is stronger (more monotonic) for Poisson kriging than for local empirical Bayes smoother since the later accounts for the local variance too; recall Figure 2. Therefore, the difference between LBS and PK kernel weights increases as the local variance increases; see Figure 11 (left bottom graph). Differences in kernel weights, however, cannot account for the differences in risk estimates (Figure 11, right bottom scattergram), suggesting that other factors, such as the spatial patterns incorporated in the computation of Poisson kriging weights but ignored in LBS, are responsible for these discrepancies.

**Figure 12:**
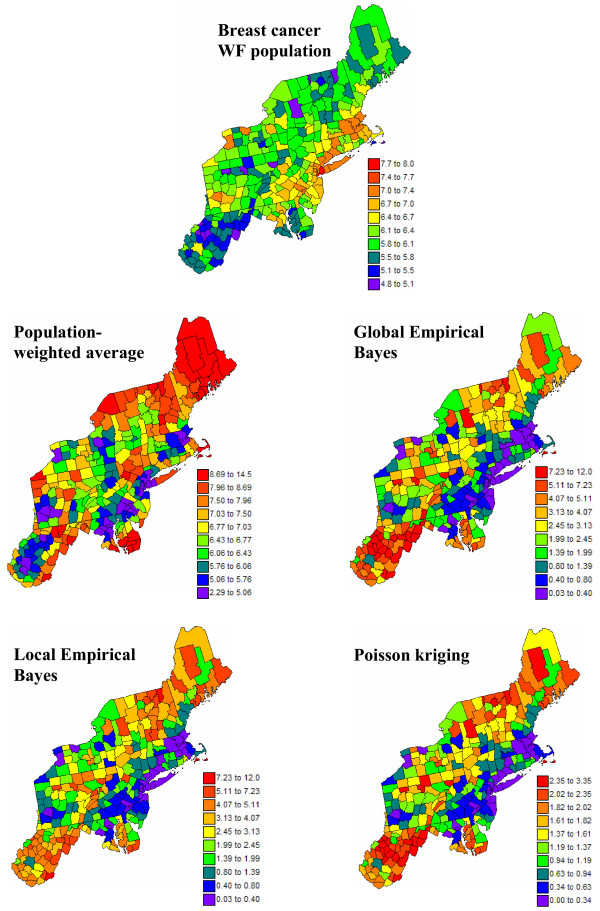
**Maps of mean square error of prediction for the breast cancer risk maps of Figure 8**. The fill color in each county represents the mean square error of prediction of the risk of breast cancer produced by the following predictors: population-weighted average, local and global empirical Bayes smoothers, and Poisson kriging. The units are age-adjusted mortality rate per 100,000 person-years, and the class boundaries correspond to the deciles of the histogram of mean square error of prediction.

**Figure 13:**
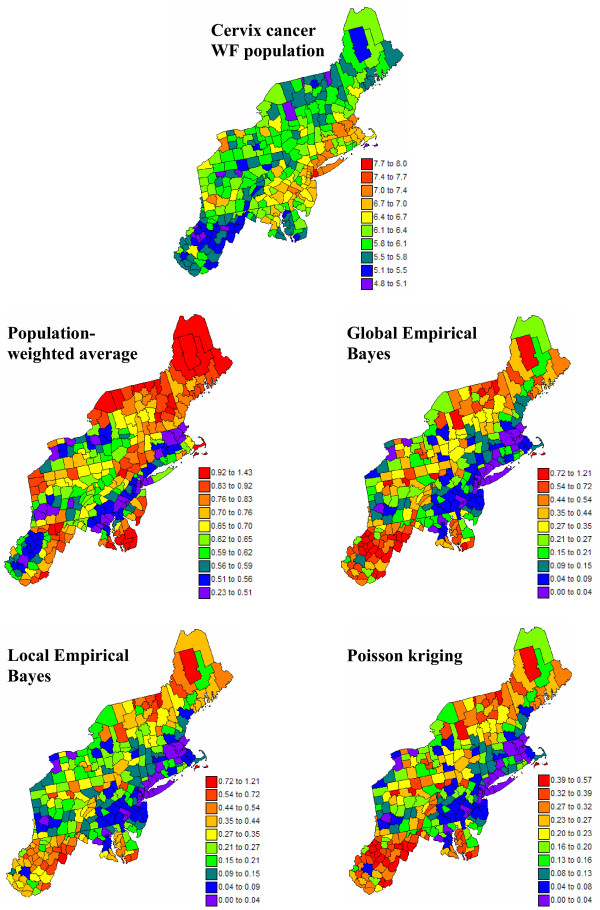
**Maps of mean square error of prediction for the cervix cancer risk maps of Figure 9**. The fill color in each county represents the mean square error of prediction of the risk of cervix cancer produced by the following predictors: population-weighted average, local and global empirical Bayes smoothers, and Poisson kriging. The units are age-adjusted mortality rate per 100,000 person-years, and the class boundaries correspond to the deciles of the histogram of mean square error of prediction.

The mean square error of prediction (i.e. prediction variance) associated with the risk maps of Figures 7 and 8 are mapped in Figures 12 and 13. Relative results are very similar for both types of cancers since the spatial distribution of population sizes and the coordinates of the county centroids control, to a large extent, the spatial pattern of the maps. The PWA map shows larger variances along the edge of the study area, that is where fewer counties are close geographically, or where the distance between centroids is larger such as in Maine. In these situations the information carried out by the neighboring counties, as measured by the spatial covariance function, is smaller, leading to a larger prediction variance. The impact of the spatial distribution of population sizes on the variance map is much more pronounced for the three other estimators: prediction variances are smaller around the major cities of the East Coast and larger in the South. The relative magnitude of the Poisson kriging variance is however much lower than the prediction variance of the empirical Bayes smoothers. Population-weighted average has the largest prediction variance.

### Simulation studies

Figures 7 through 13 illustrated the major differences between alternative approaches for correction of the small number problem. An objective assessment of the prediction performances of the various techniques requires, however, the availability of the underlying risk maps which are unknown in practice. Simulation provides a way to generate multiple realizations of the spatial distribution of cancer mortality rates under specific scenarios for the underlying risk and population sizes. Predicted risks can then be compared to the risk maps used in the simulation. Marshall [14] used a similar approach to investigate the performances of local and global empirical Bayes estimators under different scenarios for the disease frequency, the size of the population at risk, and the spatial patterns of risk. More recently, Richardson *et al. *[36] used simulation to investigate the performance of various disease-mapping models for recovering the "true" risk surfaces, in particular the ability to detect risk-raised areas.

In this paper, a series of simulated maps of age-adjusted breast and cervix cancer mortality rates {z^(l)^(**u**_α_), α = 1,...,N} were generated in order to investigate the prediction performance of the four methods implemented in **poisson_kriging.exe**. For each cancer, three different scenarios in terms of the underlying risk map {r(**u**_α_), α = 1,...,N} were considered:

1) map of risk estimated from mortality rates by Poisson kriging,

2) smooth map of regional risk estimated from mortality rates by a factorial kriging variant of Poisson kriging (right-hand side covariance terms in the first K equations of system (16) are set to zero; see analogous approach in [22]),

3) non-structured map of risk created by random shuffling of the risk values estimated by Poisson kriging.

**Figure 14:**
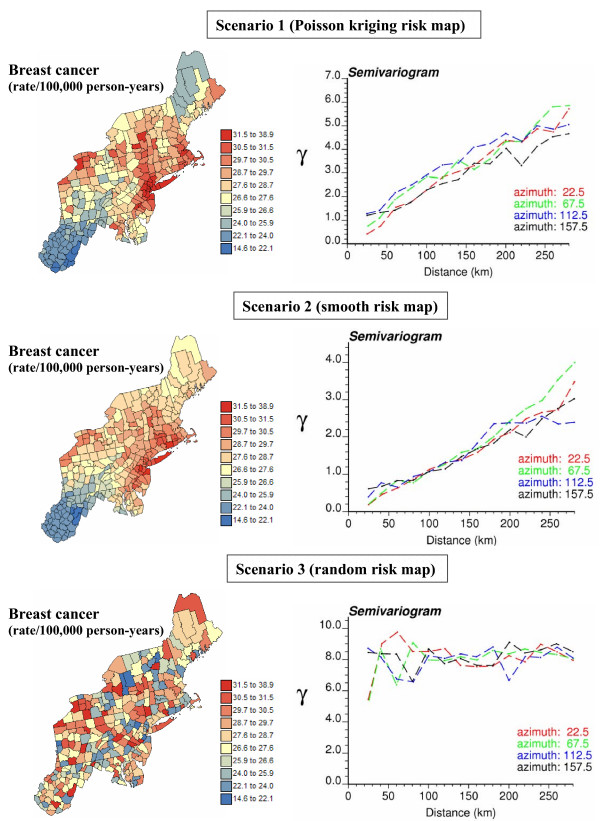
**Breast cancer risk maps (3 scenarios) used in the simulation procedure, and the corresponding directional semivariograms**. The fill color in each county represents the mortality risk over the period 1970–1994 (age-adjusted mortality rate per 100,000 person-years). Three scenarios are considered for the risk values: risk estimated from the age-adjusted mortality rates of Figure 1 by Poisson kriging, smooth map of regional risks, and non-structured map of risk created by random shuffling of the scenario 1 map.

The three risk maps for breast cancer, with the corresponding semivariograms, are displayed in Figure 14. At this stage, Poisson kriging was merely used to create risk maps with various degree of smoothness; simple population-weighted averages would have generated similar results. Poisson kriging is however not used to generate simulated values (see below), hence the results of the performance comparison are not biased by the way the risk maps were created.

**Figure 15:**
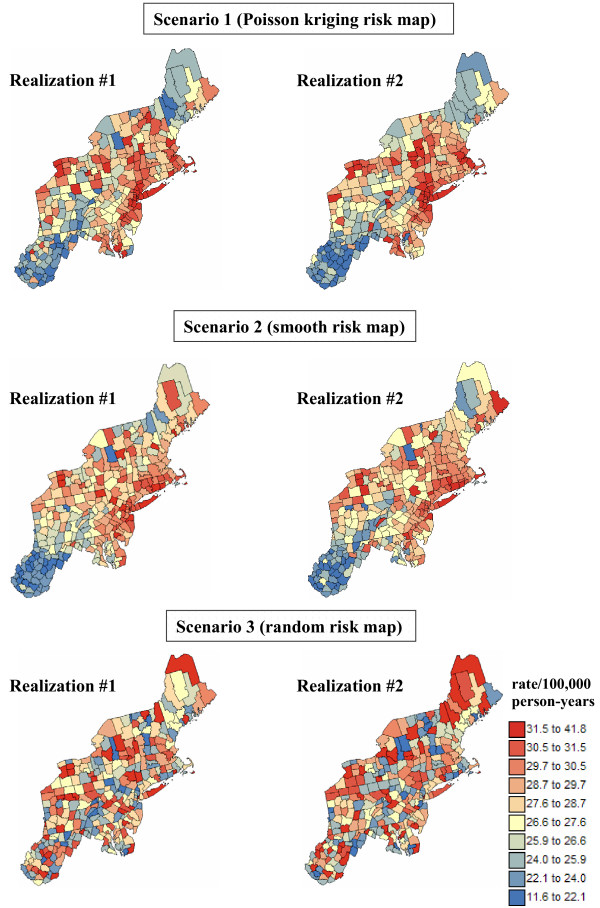
**Maps of breast cancer mortality rates simulated under three scenarios for the underlying risk map**. The number of cases for each county was simulated by random sampling of a Poisson distribution that is defined by the risk and population value derived from the three risk maps of Figure 14 and the white female population map of Figure 1. The units are age-adjusted mortality rates per 100,000 person-years. The color legend applies to all the maps; the class boundaries correspond to the deciles of the histogram of original rates.

For each cancer and each risk map, 100 realizations of the number of cases recorded over each county with centroid **u**_α _was generated by random drawing of a Poisson distribution whose mean parameter is r(**u**_α_) × n(**u**_α_). Both white and black female population maps of Figure 1 were used in the simulation, leading to six different scenarios (3 risk maps × 2 population maps) for each cancer. Figure 15 shows the first two realizations of breast cancer mortality rates generated using the risk maps of Figure 14 and the white female population map displayed in Figure 1.

### Comparison of prediction performances

Each map of simulated rates {z^(l)^(**u**_α_), α = 1,...,N} underwent a (geo)statistical analysis similar to the one implemented in **poisson_kriging.exe**. The six following predictors of risk values were used:

1. Observed rate

2. Population-weighted average

3. Global empirical Bayes smoother

4. Local empirical Bayes smoother

5. Poisson kriging using the 'true' risk semivariogram (i.e. traditional semivariogram computed from the underlying risk values)

6. Poisson kriging using the risk semivariogram estimated from observed rates using expression (18).

Estimators 2 through 6 were based on the closest 32 recorded rates.

Predicted risks { (**u**_α_), α = 1,...,N} and the corresponding prediction variance {[ (**u**_α_)]^2^, α = 1,...,N} were compared to the underlying risk map {r(**u**_α_), α = 1,...,N}. Various performance criteria were computed and averaged over all 100 realizations to attenuate the impact of statistical fluctuations.

#### Bias and accuracy of prediction

The first two criteria are the mean error (ME) and mean square error (MSE) of prediction computed as:





Table 2 indicates that, on average over 100 realizations, the prediction using the observed rates is the least biased, which is expected since these rates were generated by random drawing of distributions centred on the risk values. For both cancers, Poisson kriging has less bias than the empirical Bayes smoothers and the population-weighted average. The worst results are obtained for global empirical Bayes smoothers, in particular when the risk values are spatially structured (scenarios 1–2). For several realizations generated using the smooth risk map and black female populations (13 realizations for breast and 6 for cervix), the global variance of simulated rates is smaller than the ratio m*/, leading to zero shrinkage factor and uniform maps of risk estimates; recall Equation (10). The same effect is observed for two breast cancer maps generated under scenario 1 (BF population). The occurrence of zero shrinkage factor, however, cannot explain the larger bias observed when global empirical Bayes smoothers are applied to simulations using the white female population maps.

**Table 2:** Performance comparison of alternative estimators: mean error of prediction. Results obtained on average over 100 realizations generated under two different population size scenarios and 3 types of risk map (1 = observed, 2 = smooth, 3 = random). Poisson kriging was conducted with the semivariogram estimated from the underlying risk values (true γ_R_(**h**)) or the simulated mortality rates. Bold numbers refer to best performances outside the ideal case where the true semivariogram of risk is known.

Estimators	WF population			BF population		
BREAST CANCER	Scenario 1	Scenario 2	Scenario 3	Scenario 1	Scenario 2	Scenario 3

Observed rates	**-0.006**	**0.004**	**-0.020**	**-0.221**	**0.141**	**-0.198**
Population-weighted average	0.996	0.491	0.303	1.654	0.720	0.562
Global Empirical Bayes	1.245	1.127	0.162	3.094	1.921	0.581
Local Empirical Bayes	0.586	0.395	0.130	1.623	0.737	0.572
Poisson kriging (true γ_R_(**h**))	0.237	0.175	0.005	1.264	0.600	0.372
Poisson kriging	0.228	0.193	0.016	1.441	0.622	0.412

CERVIX CANCER						

Observed rates	**0.000**	**0.001**	**-0.003**	0.248	**0.059**	**-0.010**
Population-weighted average	-0.362	-0.196	-0.139	-0.406	-0.261	-0.270
Global Empirical Bayes	-0.386	-0.398	-0.092	-0.717	-0.697	-0.337
Local Empirical Bayes	-0.184	-0.142	-0.066	-0.331	-0.215	-0.203
Poisson kriging (true γ_R_(**h**))	-0.051	-0.024	-0.034	-0.299	-0.198	-0.197
Poisson kriging	-0.049	-0.019	-0.028	**-0.221**	-0.118	-0.167

The benefit of using predicted risks over recorded mortality rates is striking when looking at the mean square error of prediction, in particular when population sizes are smaller. Whenever the risk is spatially structured, Table 3 shows that Poisson kriging using the true semivariogram model outperforms all other methods. The use of the estimated risk semivariogram slightly increases PK prediction errors, making it a close second to the population-weighted average for the analysis of cervix cancer in smaller populations (BF). Global empirical Bayes smoother leads to the smallest MSE values when the underlying risk is spatially unstructured. This result is in agreement with simulation experiments by Marshall [14] that demonstrated better prediction performances of global versus local Bayes estimators when the underlying spatial pattern of the disease is random.

**Table 3:** Performance comparison of alternative estimators: mean square error of prediction. Results obtained on average over 100 realizations generated under two different population size scenarios and 3 types of risk map (1 = observed, 2 = smooth, 3 = random). Poisson kriging was conducted with the semivariogram estimated from the underlying risk values (true γ_R_(**h**)) or the simulated mortality rates. Bold numbers refer to best performances outside the ideal case where the true semivariogram of risk is known.

Estimators	WF population			BF population		
BREAST CANCER	Scenario 1	Scenario 2	Scenario 3	Scenario 1	Scenario 2	Scenario 3

Observed rates	4.426	4.462	4.777	1178	1461	1321
Population-weighted average	3.249	1.085	8.159	8.093	3.753	11.40
Global Empirical Bayes	5.090	4.591	**2.286**	17.09	9.536	**6.853**
Local Empirical Bayes	1.514	0.811	2.424	8.631	5.543	11.02
Poisson kriging (true γ_R_(**h**))	0.828	0.593	2.376	6.154	3.534	9.648
Poisson kriging	**0.857**	**0.625**	2.452	**7.107**	**3.741**	10.03

CERVIX CANCER						

Observed rates	0.803	0.802	0.738	367	257	232
Population-weighted average	0.590	0.149	1.006	**1.088**	**0.617**	1.468
Global Empirical Bayes	0.556	0.496	**0.318**	1.387	1.079	**0.906**
Local Empirical Bayes	0.264	0.116	0.341	3.143	1.685	3.440
Poisson kriging (true γ_R_(**h**))	0.177	0.041	0.313	0.881	0.566	1.233
Poisson kriging	**0.179**	**0.045**	0.335	1.117	0.764	1.383

#### Detection of zones of low and high risks

Cancer mortality maps are frequently used by public health officials to identify areas of excess and to guide surveillance and control activities. It is thus important that the prediction method lead to a correct ranking of geographical units in terms of mortality risk. The Spearman rank correlation coefficient measures the strength of the monotonic relation between two variables. For each *l*-th realization, the correlation between the rank of the actual and estimated risk values was computed as:



where  (**u**_α_) and y(**u**_α_) are the rank of the estimated and actual risk values,  (**u**_α_) and r(**u**_α_), in their respective distributions. The corresponding mean and standard deviation are denoted  and *s*. The rank correlation was averaged over all realizations, and their values for the different scenarios are listed in Table 4. As for the mean square error of prediction in Table 3, Poisson kriging generally yields the largest rank correlation for spatially structured risk maps, while global empirical Bayes smoother performs the best when the underlying risk is spatially random. An interesting result is the lack of correlation between true risk values and population-weighted averages under scenario 3. The smoothing effect caused by the very small PWA kernel weight (recall Table 1) leads to estimated risk maps that display spatial correlation even when the underlying risk map is purely random. In other words, the application of a moving average to a purely random field creates a field of spatially structured values. To attenuate PWA smoothing the number of closest neighbors was reduced from K = 32 to K = 16. The smaller averaging window generated less smoothing and slightly larger rank correlations; e.g., ρ_rank _= 0.218 instead of 0.047 for WF population, Scenario 3. Yet, the use of fewer observations led to larger prediction errors when population sizes are smaller; e.g. for breast cancer, MSE = 17.89 instead of 11.40 for BF population (Scenario 3) or MSE = 12.44 instead of 8.093 for Scenario 1. A similar increase in prediction errors occurred for local empirical Bayes smoother and Poisson kriging. The parameter K = 32 was thus kept throughout the analysis.

**Table 4:** Performance comparison of alternative estimators: rank correlation coefficient between estimates and true risk values. Results obtained on average over 100 realizations generated under two different population size scenarios and 3 types of risk map (1 = observed, 2 = smooth, 3 = random). Poisson kriging was conducted with the semivariogram estimated from the underlying risk values (true γ_R_(**h**)) or the simulated mortality rates. Bold numbers refer to best performances outside the ideal case where the true semivariogram of risk is known.

Estimators	WF population			BF population		
BREAST CANCER	Scenario 1	Scenario 2	Scenario 3	Scenario 1	Scenario 2	Scenario 3

Observed rates	0.813	0.761	0.805	0.355	0.328	0.199
Population-weighted average	0.792	0.899	0.047	0.623	**0.737**	-0.001
Global Empirical Bayes	0.751	0.720	**0.834**	0.129	0.146	**0.363**
Local Empirical Bayes	0.896	0.922	0.825	0.612	0.708	0.217
Poisson kriging (true γ_R_(**h**))	0.930	0.927	0.822	0.699	0.744	0.257
Poisson kriging	**0.929**	**0.925**	0.818	**0.654**	0.732	0.225

CERVIX CANCER						

Observed rates	0.765	0.682	0.770	-0.191	-0.282	0.101
Population-weighted average	0.706	0.886	0.006	0.445	**0.650**	-0.017
Global Empirical Bayes	0.748	0.664	**0.811**	0.314	0.225	**0.354**
Local Empirical Bayes	0.867	0.906	0.789	0.479	0.598	0.189
Poisson kriging (true γ_R_(**h**))	0.905	0.967	0.807	0.593	0.676	0.228
Poisson kriging	**0.903**	**0.961**	0.790	**0.558**	0.611	0.200

#### Quality of the uncertainty model

The ability of the prediction variance to capture the actual magnitude of the prediction error was quantified using the following mean square standardized residual (MSSR):



If the actual estimation error is equal, on average, to the error predicted by the model, the MSSR statistic should be about one [[37], p. 91]. Using m*/n(**u**_α_) as an estimate of the prediction variance for the observed rates z(**u**_α_) leads to the best MSSR statistic for half the scenarios; see Table 5. However, this result simply indicates that one correctly predicts that observed rates fare badly in estimating the underlying risk (recall Table 3). Ignoring the results for observed rates, Poisson kriging slightly outperforms the local empirical Bayes smoother. For small populations, the prediction variance for population-weighted averages and empirical Bayes smoothers systematically overestimates the actual magnitude of prediction errors.

**Table 5:** Performance comparison of alternative estimators: mean square standardized residual. Results obtained on average over 100 realizations generated under two different population size scenarios and 3 types of risk map (1 = observed, 2 = smooth, 3 = random). Poisson kriging was conducted with the semivariogram estimated from the underlying risk values (true γ_R_(**h**)) or the simulated mortality rates. Bold numbers refer to best performances outside the ideal case where the true semivariogram of risk is known.

Estimators	WF population			BF population		
BREAST CANCER	Scenario 1	Scenario 2	Scenario 3	Scenario 1	Scenario 2	Scenario 3

Observed rates	**0.913**	**0.948**	0.983	**0.891**	0.948	**0.980**
Population-weighted average	0.817	0.735	1.076	0.473	0.260	0.624
Global Empirical Bayes	1.524	1.974	0.973	0.494	0.284	0.315
Local Empirical Bayes	0.823	0.799	**1.011**	0.519	0.318	0.667
Poisson kriging (true γ_R_(**h**))	0.929	0.986	1.151	2.324	1.705	1.447
Poisson kriging	0.901	1.436	1.197	2.365	**0.947**	2.066

CERVIX CANCER						

Observed rates	1.224	**1.188**	1.050	1.299	**1.247**	**1.096**
Population-weighted average	1.229	0.542	1.156	0.441	0.257	0.540
Global Empirical Bayes	1.506	1.476	**1.028**	0.354	0.248	0.289
Local Empirical Bayes	1.154	0.679	1.101	0.638	0.407	0.663
Poisson kriging (true γ_R_(**h**))	1.135	1.053	1.032	1.184	0.754	1.039
Poisson kriging	**1.071**	0.760	1.252	**0.880**	0.600	1.179

Another way to use the prediction variance is to build at any location **u**_α _the conditional cumulative distribution function (ccdf) of the unknown risk value. Under the assumption of normality of the prediction errors, the ccdf is defined as:



where *G*(·) is the cumulative distribution function of the standard normal distribution. The ccdf allows one to compute the probability that the risk variable does not exceed any specific threshold *r *over the entity with centroid **u**_α_. This distribution is here fully characterized by its mean and variance which are the risk estimate, (**u**_α_) and the prediction variance, [ (**u**_α_)]^2^. From the ccdf one can compute a series of symmetric *p*-probability intervals (PI) bounded by the (1-*p*)/2 and (1+*p*)/2 quantiles of that function. For example, the 0.5-PI is bounded by the lower and upper quartiles [ (**u**_α_;0.25|(Info)),  (**u**_α_;0.75|(Info))]. A correct modeling of local uncertainty would entail that there is a 0.5 probability that the actual risk value at **u**_α _falls into that interval or, equivalently, that over the study area 50% of the 0.5-PI include the true value. In our simulation studies the true risk values are known, hence from the independently derived ccdfs at the *N *locations **u**_α _one can compute the fraction of true values falling into the symmetric *p*-PI as:



where ζ^(*l*) ^(**u**_α_; *p*) equals 1 if *r*(**u**_α_) lies between the (1-*p*)/2 and (1+*p*)/2 quantiles of the ccdf for the l-th realization, and zero otherwise. The scattergram of the estimated,  (*p*), versus expected, *p*, fractions is called the "accuracy plot". Deutsch [38] proposed to assess the closeness of the estimated and theoretical fractions using the following "goodness" statistic:



where *w*(*p*_*k*_) = 1 if  (*p*_*k*_) > *p*_*k*_, and 2 otherwise. Twice more importance is given to deviations when  (*p*_*k*_) <*p*_*k *_(inaccurate case), i.e. the case where the fraction of true values falling into the *p*-PI is smaller than expected. K' represents the discretization level of the computation; for example, the ccdf percentiles are used as PI boundaries when K' = 50. Table 6 indicates that no technique systematically outperforms the others. Empirical Bayes smoothers are best for breast cancer, while Poisson kriging performs better for the less common cervix cancer.

**Table 6:** Performance comparison of alternative estimators: goodness of the model of uncertainty. Results obtained on average over 100 realizations generated under two different population size scenarios and 3 types of risk map (1 = observed, 2 = smooth, 3 = random). Poisson kriging was conducted with the semivariogram estimated from the underlying risk values (true γ_R_(**h**)) or the simulated mortality rates. Bold numbers refer to best performances outside the use of observed rates and the ideal case where the true semivariogram of risk is known.

Estimators	WF population			BF population		
BREAST CANCER	Scenario 1	Scenario 2	Scenario 3	Scenario 1	Scenario 2	Scenario 3

Observed rates	0.973	0.974	0.975	0.956	0.964	0.965
Population-weighted average	**0.967**	0.943	0.968	0.887	0.761	0.910
Global Empirical Bayes	0.847	0.843	**0.974**	**0.908**	0.787	0.804
Local Empirical Bayes	0.964	**0.952**	0.971	0.899	0.782	**0.921**
Poisson kriging (true γ_R_(**h**))	0.971	0.972	0.947	0.716	0.842	0.887
Poisson kriging	0.965	0.913	0.940	0.761	**0.927**	0.803

CERVIX CANCER						

Observed rates	0.937	0.939	0.970	0.935	0.935	0.922
Population-weighted average	0.933	0.901	0.933	0.852	0.743	0.888
Global Empirical Bayes	0.875	0.870	**0.973**	0.833	0.785	0.787
Local Empirical Bayes	0.960	0.920	0.962	0.875	0.771	0.901
Poisson kriging (true γ_R_(**h**))	0.963	0.947	0.973	0.931	0.935	0.966
Poisson kriging	**0.968**	**0.924**	0.934	**0.940**	**0.893**	**0.935**

Not only should the true value fall into the PI according to the expected probability *p*, but this interval should be as narrow as possible to reduce the uncertainty about that value. In other words, among two probabilistic models with similar goodness statistics one would prefer the one with the smallest spread (less uncertain). In this paper the ccdf spread is quantified by its variance and averaged over all 295 counties, leading to the following statistic:



Table 7 shows that, regardless the type of cancer, the PK ccdf variance is the smallest for all scenarios. Consequently, the probability intervals are narrower and more informative if they include the expected fraction of true values.

**Table 7:** Performance comparison of alternative estimators: spread of the model of uncertainty. Results obtained on average over 100 realizations generated under two different population size scenarios and 3 types of risk map (1 = observed, 2 = smooth, 3 = random). Poisson kriging was conducted with the semivariogram estimated from the underlying risk values (true γ_R_(**h**)) or the simulated mortality rates. Bold numbers refer to best performances outside the ideal case where the true semivariogram of risk is known.

Estimators	WF population			BF population		
BREAST CANCER	Scenario 1	Scenario 2	Scenario 3	Scenario 1	Scenario 2	Scenario 3

Observed rates	5.147	5.002	4.784	22482	21577	20641
Population-weighted average	4.057	1.603	7.712	17.60	16.28	20.97
Global Empirical Bayes	2.438	1.681	2.372	31.74	30.42	27.65
Local Empirical Bayes	2.050	1.143	2.422	16.98	16.53	18.88
Poisson kriging (true γ_R_(**h**))	0.940	0.603	2.076	2.456	1.890	6.621
Poisson kriging	**1.042**	**0.470**	**2.074**	**8.775**	**6.663**	**5.987**

CERVIX CANCER						

Observed rates	0.586	0.608	0.684	2359	2372	2625
Population-weighted average	0.494	0.242	0.883	2.870	2.631	3.233
Global Empirical Bayes	0.293	0.259	0.317	4.242	4.327	4.148
Local Empirical Bayes	0.229	0.170	0.307	2.843	2.722	3.130
Poisson kriging (true γ_R_(**h**))	0.155	0.043	0.307	0.763	0.711	1.201
Poisson kriging	**0.169**	**0.069**	**0.269**	**1.552**	**1.825**	**1.529**

#### Smoothing effect

All prediction methods are based on linear combinations of surrounding rates; hence they are all expected to create risk maps that are smoother than the original map of observed rates. One should however avoid any over smoothing that could potentially lead one to overlook the presence of high risk or low risk areas. The variance of the risk estimates, denoted , was computed for each realization and the averages over 100 realizations are listed in Table 8. Global empirical Bayes smoother, and population-weighted average for random risk maps, generate the largest smoothing effect. For the white female population, PK risk estimates display the largest variance and the one that is the closest to the variance of the underlying risk, . For simulations based on smaller population sizes the largest variance are obtained using local empirical Bayes smoothers but the true risk variance is severely overestimated, in particular for the less common cervix cancer. Population-weighted averages and Poisson kriging perform equally well in this situation.

**Table 8:** Performance comparison of alternative estimators: variance of risk estimates. Results obtained on average over 100 realizations generated under two different population size scenarios and 3 types of risk map (1 = observed, 2 = smooth, 3 = random). Poisson kriging was conducted with the semivariogram estimated from the underlying risk values (true γ_R_(**h**)) or the simulated mortality rates. Bold numbers refer to best performances (i.e. the variance of risk estimates is the closest to the true risk variance reported in the first row) outside the ideal case where the true semivariogram of risk is known.

Estimators	WF population			BF population		
BREAST CANCER	Scenario 1	Scenario 2	Scenario 3	Scenario 1	Scenario 2	Scenario 3

True risk values	7.891	5.982	7.891	7.891	5.982	7.891
Population-weighted average	5.635	4.560	0.346	8.538	**6.881**	3.035
Global Empirical Bayes	3.628	1.722	5.178	0.290	0.145	1.026
Local Empirical Bayes	6.571	4.941	5.257	9.364	8.576	**5.339**
Poisson kriging (true γ_R_(**h**))	6.792	5.176	5.561	8.156	6.781	4.161
Poisson kriging	**6.922**	**5.093**	**5.498**	**8.332**	7.044	4.143

CERVIX CANCER						

True risk values	0.946	0.596	0.946	0.946	0.596	0.946
Population-weighted average	0.485	0.498	0.055	**0.909**	**0.918**	0.422
Global Empirical Bayes	0.474	0.213	0.593	0.132	0.050	0.162
Local Empirical Bayes	0.727	0.538	**0.625**	3.197	2.059	2.746
Poisson kriging (true γ_R_(**h**))	0.793	0.599	0.671	0.941	0.910	0.550
Poisson kriging	**0.804**	**0.596**	0.607	1.269	1.137	**0.690**

#### Spread of realizations into the multivariate space of performance criteria

A limitation of the analysis detailed in Tables 2 through 8 is that each performance criterion is considered separately and its value is averaged over all 100 realizations. Information about the spread of results among realizations, as well as the correlation (redundancy) between criteria, is lost as long as one does not look at the scatter of realizations in the seven-dimensional space spanned by the seven performance criteria. Principal component analysis (PCA) was used to display the 500 realizations (5 predictors × 100 realizations) in a subspace that can be visualized easily. The basic idea of PCA is to create new orthogonal variables, the principal components, as linear combinations of the original variables, i.e. the 7 performance criteria [37]. The first few components account for most of the variance and so are the most informative. In this paper the first two components, which account for 64 to 97% of the global variance depending on the scenario and cancer type, were computed. These components were rotated (Varimax rotation) to achieve a "simple structure", that is each performance criterion correlates mainly with one of the two principal components, which facilitates the interpretation of the components [39,40].

**Figure 16:**
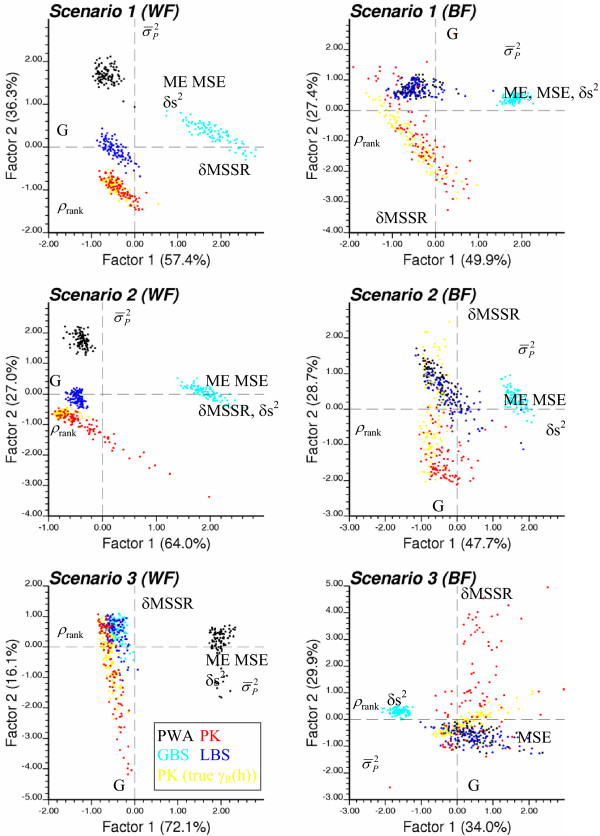
**Scatter of realizations in the multivariate space of performance criteria (breast cancer)**. For each risk and population scenario, a principal component analysis is conducted on the correlation matrix of the seven performance criteria, and individual realizations are projected in the space of the first two rotated components. The estimators are: population-weighted mean (black), global (light blue) and local (dark blue) empirical Bayes smoothers, and Poisson kriging using the true semivariogram of risk (yellow) or the one modelled from observed mortality rates (red). The position of criteria labels on the graph indicates their correlation with the two principal components (criteria with small correlations are not displayed).

**Figure 17:**
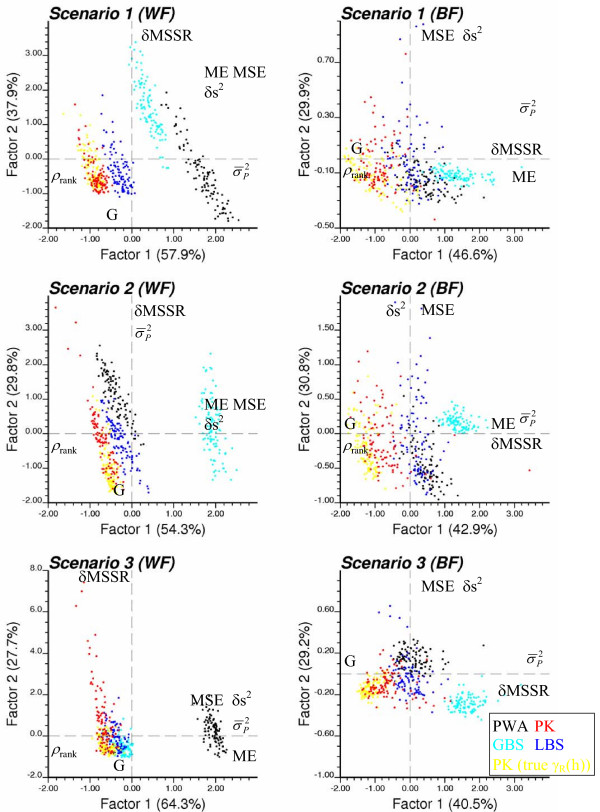
**Scatter of realizations in the multivariate space of performance criteria (cervix cancer)**. For each risk and population scenario, a principal component analysis is conducted on the correlation matrix of the seven performance criteria, and individual realizations are projected in the space of the first two rotated components. The estimators are: population-weighted mean (black), global (light blue) and local (dark blue) empirical Bayes smoothers, and Poisson kriging using the true semivariogram of risk (yellow) or the one modelled from observed mortality rates (red). The position of criteria labels on the graph indicates their correlation with the two principal components (criteria with small correlations are not displayed).

Figures 16 and 17 show the scatter of realizations under each of the six scenarios for breast and cervix cancers, respectively. The performance criteria  and MSSR were replaced by δs^2 ^= | - | and δMSSR = |MSSR-1|, so that each criterion needs either to be minimized (ME, MSE, δMSSR, δs^2^, ) or maximized (G, ρ_rank_). Note that the upperscript "^(*l*)^" for realization is dropped to simplify the notation. The position of the criterion labels on each plot in Figures 16 and 17 indicates the correlation between the performance criteria and the principal components. Typically, one component captures the negative correlation between the rank correlation coefficient ρ_rank _and the magnitude of the prediction error (i.e. ME, MSE). The other component depicts the negative correlation between the goodness statistic G and the standardized residual statistic δMSSR. In all cases, the best predictors are located in the lower left quadrant of the graph, corresponding to negative values for both rotated principal components (i.e. larger goodness statistic and rank correlation).

For breast cancer the horizontal axis can be interpreted as the magnitude of prediction errors, leading to a clear separation of GBS realizations (light blue) from other predictors for structured risk patterns (scenarios 1 and 2) where the global empirical Bayes smoother performs badly. For the random risk maps, PWA results (black) are the worst for white female populations while GBS performs the best for smaller population sizes. The vertical axis allows one to better discriminate the approaches that have similar prediction errors. Clearly, Poisson kriging yields the best predictions for white female populations when the risk is spatially structured and for black female populations when the risk varies smoothly in space. There is more overlap between predictors for the other scenarios. In particular, the PWA and LBS clouds coincide for all three scenarios under smaller population sizes. These graphs also highlight the larger scatter of PK realizations (red), which is very pronounced when the risk map is random. This variability is mainly caused by the estimation and modelling of the risk semivariogram, since Poisson kriging using the true semivariogram model (yellow) leads to more compact clouds.

Most of the conclusions drawn from the analysis of breast cancer results are confirmed by the principal component analysis of cervix cancer scores. Figure 17 shows that Poisson kriging performs better than other approaches for most scenarios, with a clear benefit when the risk values are spatially correlated. Once again, the PWA and LBS clouds overlap for all three scenarios under smaller population sizes.

## Conclusion

Cancer mortality maps are used by public health officials to identify areas of excess and to guide surveillance and control activities. Quality of decision-making thus relies on an accurate quantification of risks from observed rates that can be very unreliable when computed from sparsely populated geographical units or recorded for minority populations. Smoothers, such as the median-based head banging or the global empirical Bayes estimator, are routinely applied to stabilize rates. Yet, these methods are unable to account for the fact that rates measured in entities that are close in the geographic space tend to be more similar than the ones recorded further apart. Deterministic approaches, such as the head-banging algorithm, also fail to provide any measure of the uncertainty attached to the predicted risks. These shortcomings are overcome by the rich class of full Bayes models, which yields the full posterior distribution of the risk while accounting for the uncertainty in the parameters of the model. However, implementation of these sophisticated methods is still cumbersome and relies on time-consuming iterative procedures, which led to the following statement by Leyland and Davies [11]: "*Given that the nonspatial empirical Bayes estimator appears to perform adequately in the presence of spatial autocorrelation, and given the range of standard statistical packages that can fit such models, we should question whether the additional information available from a full Bayes model (the full posterior distribution) is always of sufficient importance to justify the added complexity of (and computational time required by) Gibbs sampling*."

The geostatistical predictor introduced in this paper, although not as straightforward as an empirical Bayes estimator, is easier to implement than a full Bayes model and does not require the distributional assumptions underlying Diggle *et al*.'s model-based kriging [27]. It allows one to model the spatial correlation of health data and incorporate the spatial dependence into the estimation of the underlying risk and the associated uncertainty. Unlike traditional semivariogram analysis and kriging, Poisson kriging do recognize that health data are comprised of a numerator and a denominator and that the semivariogram of unknown risk values cannot be simply equaled to the semivariogram of observed rates. The trade-off cost for the simplicity of Poisson kriging is that, unlike the full Bayesian approach, the uncertainty attached to the parameters of the correlation function is ignored in the analysis, which should lead to smaller prediction variances in general [16]. Apart from one case study in ecology [26], the prediction performances of Poisson kriging and complex Bayesian models have not been compared yet, and detailed simulation studies under various conditions should be conducted in the future. Based on Monestiez et al.'s experience that a full Bayesian approach is more than 500 times slower than Poisson kriging [16], the simulation study presented in this paper would however require about one year of Cpu time.

The analysis of age-adjusted breast and cervix cancer mortality rates illustrated some key features of population-weighted, empirical Bayes and Poisson kriging estimators. Because of the small weight assigned to the rate recorded over the entity being smoothed (kernel weight), population-weighted average leads to an over smoothing of mortality maps. This smoothing could be reduced by decreasing the number of closest neighbors (K = 32 in the analysis) but at the expense of larger prediction errors for smaller population sizes. The other techniques assign larger and similar kernel weights but they use a different piece of auxiliary information in the prediction: global or local means for global or local empirical Bayes smoothers, and spatial combination of surrounding rates for Poisson kriging. Results are thus influenced by changes in local statistics (mean and variance) of mortality rates across the study area, as well as their spatial pattern which includes the distance of autocorrelation, the importance of short-range fluctuation (nugget effect), and the presence of direction-dependent variability (anisotropy). As the population size decreases, the kernel weight decreases, enhancing the importance of this auxiliary information and so differences among methods.

Simulation studies allowed a quantitative assessment of the performance of various smoothers under different scenarios for the disease frequency, the population size, and the spatial pattern of risk. Unlike many studies in the literature, the current analysis considered non only how well the underlying risk is predicted, but also how the magnitude of the actual prediction error is correctly assessed by the uncertainty measure attached to the prediction. By analogy with the computation of the kriging variance, simple formulas were proposed to approximate the mean square error of prediction by population-weighted averages and empirical Bayes smoothers. The analysis of mean square standardized residuals showed that these prediction variances provide reasonable measures of the magnitude of prediction errors for white populations, while being too conservative for smaller population sizes.

Principal component analysis was used to visualize results in the multidimensional space of the performance criteria. Poisson kriging performs better than other approaches for most scenarios, with a clear benefit when the risk values are spatially correlated. Global empirical Bayes smoothers provide more accurate predictions when the risk is spatially random, which might not be a common situation. Because of its algorithmic complexity, the NCI head-banging approach was not coded for the simulation studies and it would have been too cumbersome to run the public-domain code for each of the 1,200 realizations individually. Analysis of a few realizations, however, showed that the method is outperformed by other methods, in particular Poisson kriging and local empirical Bayes smoother.

The implementation of the developed methodology was facilitated by the initial assumption that all geographical units are the same size, which allowed the use of geographical centroids in semivariogram estimation and kriging. This assumption is unsatisfactory when working with vastly different entities, such as SEA units over the US. A proper account of the spatial support would also allow the mapping of the risk within each unit. This issue is the topic of current research and will capitalize on recent work in the area of change of support and disaggregation procedures [41-43].

Arguably, one of the biggest problem facing spatial epidemiology and exposure assessment is that of combining, in a coherent way, data measured on very different supports and with different levels of reliability. Uncertainty arising from estimation of disease rates over small population sizes, as well as the uncertainty attached to the interpolation of exposure data measured at a limited number of monitoring stations, need to be properly accounted for in the analysis. The methodology presented in this paper allows the incorporation of population size and pattern of spatial dependence in the geostatistical processing of health data, thereby enabling researchers to estimate the risk and the associated uncertainty at different scales and to incorporate this assessment in local cluster analysis and exploration of relationships with socio-demographic and environmental factors. Issues, such as uncertainty propagation or analysis of scale-dependent correlation between cancer rates and covariates, will be further developed in future papers to appear in this series on the geostatistical analysis of disease data.

## Competing interests

The author is affiliated with BioMedware a research company that also develops software for the exploratory spatial and temporal analysis of health and environmental data. With funding from the National Cancer Institute, the author developed STIS (Space-Time Intelligence System), which is a commercial product of Terraseer and should include a Poisson kriging function in a future release.

## Supplementary Material

Additional File 1 Executable to conduct a geostatistical analysis of health data. Click here for file

Additional File 2 Input data file for poisson_kriging.exe. This dataset follows the Geo-EAS format and includes, for 295 counties of New England, the following information: FIPS code, spatial coordinates of the county geographic centroid, age-adjusted cancer mortality rate per 100,000 person-years, and the population at risk for the 1970–1994 period. Click here for file

Additional File 3 Input parameter file for poisson_kriging.exe. This text file includes all the variables and names of input/output files required by the program, as well as the parameters for semivariogram modelling and Poisson kriging. Click here for file
